# Utility of massive open online courses (MOOCs) concerning outbreaks of emerging and reemerging diseases

**DOI:** 10.12688/f1000research.12639.2

**Published:** 2017-12-27

**Authors:** Guido Bendezu-Quispe, Junior Smith Torres-Roman, Brenda Salinas-Ochoa, Akram Hernández-Vásquez

**Affiliations:** 1 Faculty of Public Health and Administration, Universidad Peruana Cayetano Heredia, Lima, Peru; 2 Unidad de Conocimiento y Evidencia (CONEVID), Universidad Peruana Cayetano Heredia, Lima, Peru; 3 Faculty of Medicine, Universidad Nacional San Luis Gonzaga, Ica, Peru; 4 School of Medicine, Universidad Peruana Cayetano Heredia, Lima, Peru; 5 Universidad Privada del Norte, Lima, Peru; 6 Grupo de Modelamiento Matemático y Simulación Numérica, Universidad Nacional de Ingeniería, Lima, Peru

**Keywords:** education, public health professional, education, distance, health education, education, medical, continuing; disease outbreaks, hemorrhagic fever, Ebola, Zika virus, chikungunya fever

## Abstract

The emergence and re-emergence of infectious diseases such as Ebola, chikungunya, and Zika increase the necessity of knowledgeable and skilled health professionals. Massive open online courses (MOOCs) arise as opportunities that allow people around the world to participate in higher education courses. A search was conducted on specialized MOOC platforms to find courses related to outbreaks, using terms included in the list of the WHO disease outbreaks from January 1st to December 31 ^st^, 2016. We found seven courses about Ebola, two about Zika, three about the dynamics of epidemics and pandemics, and only one course about dengue, chikungunya, and malaria. Most of the courses were conducted in English. The courses on Ebola, Zika and chikungunya were released after their last outbreak. MOOCs could be used to learn about health issues of global relevance, and with the necessity of fast divulgation of knowledge and skills. Translating the courses into more languages could give these courses more traction, and allow participation of professionals in regions affected by these outbreaks.

## Introduction

The emergence and re-emergence of infectious diseases is partly due to the climate change, more specifically due to the rise in global temperature, as well as the increased migration and unplanned urbanization ^1^. These events of great relevance to global health have turned these unknown diseases into realities many health professionals have to face daily.

Ebola virus causes an acute and severe disease that is usually fatal if left untreated. Its last outbreak in March 2014 was the largest in history, causing a dramatic number of more than 11,000 deaths and 28,000 new infections worldwide. It affected several countries in West Africa, generating much concern worldwide due to its estimated 50% mortality rate. Similarly, diseases such as chikungunya ^2^ and Zika ^3^ have shown several reasons to be considered serious infectious diseases.

Given the pandemic potential of these viral diseases, it is important to assess the knowledge and awareness of our health professionals regarding the mode of transmission of these diseases. In this era of globalization and technology, one of the main impacts of the Internet and the web 2.0 have been the acceleration of the process of sharing information, allowing health professionals to have quick and easy access to the latest research in medicine and health ^4^.

Massive open online courses (MOOCs) are online classes or lectures accessible for people all around the world that want to participate in higher education courses. MOOCs material includes videos, slideshows, discussion boards, quizzes, audios or any combination thereof. Usually, participants do not pay any fee to take a course. The topics in MOOCs vary widely and include science, engineering, and arts; and are usually developed by well-known figures in the study area ^5^.

## Methods

From 1 ^st^ May to 31 ^st^ May, 2017, we conducted a manual search on several learning platforms that offer MOOCs, including Coursera, edX, FutureLearn, Udacity, Miríada X, Alison, FUN.MOOC, Canvas Network, and Iversity to find courses about disease outbreaks using the terms included in the list of WHO disease outbreaks from January 1st to December 31st, 2016 ( Box 1). Information about the learning platform, institution, course length, time required per week, language and subtitles availability for every course were collected and reported using frequencies in the case of categorical data and ranges for numerical data. If the information about the course was not available on the learning platform that originally offered it, we use the information provided by MOOC aggregator platforms such as Class Central and MOOC List

**Box 1. List of terms included in the manual search for MOOCs about disease outbreaks, 2016**

epidemic(s), pandemic(s), outbreak(s), emerging diseases, re-emerging diseases, Ebola virus disease, Ebola virus, Ebola, ébola, EVD, viral hemorrhagic fevers, hemorrhagic fever syndrome, Zika, Zika virus, Chikungunya, Chikungunya virus, Lassa, Lassa Virus, MERS-CoV, Middle East respiratory syndrome coronavirus, Yellow fever, Lassa fever, Human infection with avian influenza, Oropouche virus, Rift Valley fever and Wild polio and vaccine derived polio

Source: WHO, Emergencies preparedness, response. Diseases outbreaks by year, 2016. http://www.who.int/csr/don/archive/year/2016/en/

## Results

We found seven courses about Ebola, two about Zika, three about the dynamics of epidemics and pandemics, and only one course about dengue, chikungunya, and malaria. The duration of the courses ranged from 2 to 10 weeks. 11 out of 13 courses were held only in English, with the possibility to select subtitles in English or other languages; there was one in English and Chinese, and only one exclusively in Spanish. Most courses (5 out of 13) originated from to USA centers including Emory University, University of Pittsburgh, The Pennsylvania State University, Harvard University and University System of Maryland. The information provided with the courses included audiovisual material, papers, and self-assessments. All the courses were made for health-related professionals and presented information about epidemiology and lessons about the outbreaks and prevention activities for a possible new scenario of transmission of infectious diseases. The courses on Ebola, Zika and chikungunya were released after the last outbreaks of these diseases, respectively ( Table 1).

**Table 1.** Characteristics of the massive open online courses (MOOCs) about disease outbreaks of 2016, offered by learning platforms.

Platform	Title	Institution (Country)	Duration in weeks	Hours per week	Language (Subtitles)
FutureLearn	Ebola in Context: Understanding Transmission, Response and Control	London School of Hygiene and Tropical Medicine (United Kingdom)	2	Self-paced	English (English)
FutureLearn	Ebola: Symptoms, History and Origins	Lancaster University (United Kingdom)	2	3	English (English)
FutureLearn	Preventing the Zika Virus: Understanding and Controlling the Aedes Mosquito	London School of Hygiene and Tropical Medicine (United Kingdom)	3	4	English (English)
Coursera	Ebola: an evolving epidemic	Emory University (USA)	6	Self-paced	English (English)
Coursera	Ebola: Vaincre ensemble!	University of Geneva (Switzerland)	5	Self-paced	French (French, English)
Coursera	Ebola: Essential Knowledge for Health Professionals	University of Amsterdam (Netherlands)	9	Self-paced	English (English, Arabic)
Coursera	In the footsteps of Zika… approaching the unknown	University of Geneva (Switzerland)	8	5	English (English, French, Portuguese, Spanish)
Coursera	Epidemics, Pandemics and Outbreaks	University of Pittsburgh (USA)	4	3–4	English (English)
Coursera	Epidemics - the Dynamics of Infectious Diseases	The Pennsylvania State University (USA)	8	2–3	English (English)
edX	Lessons from Ebola: Preventing the Next Pandemic	Harvard University (USA)	4	3–4	English (English)
edX	Global Health – The Lessons of Ebola	University System of Maryland (USA)	6	2–3	English (English)
edX	Epidemics	The University of Hong Kong (Hong Kong)	10	2–3	English, Chinese (English, Chinese)
edX	ETV: Paludismo, Dengue y Chikungunya	The Ministry of Education of Mexico (Mexico)	2	10	Spanish (Spanish)

Data on 2016 massive open online courses about disease outbreaks, offered on learning platforms, retrieved from manual searches The dataset contains information on the learning platform, institution, course length, time required per week, language and availability of subtitles. Click here for additional data file. Copyright: © 2017 Bendezu-Quispe G et al. 2017 Data associated with the article are available under the terms of the Creative Commons Zero "No rights reserved" data waiver (CC0 1.0 Public domain dedication).

## Discussion

Finding MOOCs about Ebola, chikungunya, and Zika after the start of their last outbreaks demonstrates the interest of institutions in offering information to the public. The vast majority of courses are offered in English, with a few having subtitles in other languages. MOOCs offer a recent and emerging form of education. There is a continuous increase in the number of courses offered in this format and, by 2015, a total of 35 million participants in 4.200 MOOCs were counted, with 8.27% of these courses corresponding to health and medicine.

The spread of diseases makes it necessary to invest in alternative methods of spreading knowledge, to improve the capabilities of health professionals in topics that affect people worldwide. MOOCs could be used to learn about health issues of global relevance, and with the necessity of fast divulgation of knowledge and skills. Because the countries most affected by these diseases do not have English as the native language, promoting the translation of content into more languages could give these courses more traction, and allow participation of professionals in regions affected by these outbreaks.

## Data availability

The data referenced by this article are under copyright with the following copyright statement: Copyright: © 2017 Bendezu-Quispe G et al.

Data associated with the article are available under the terms of the Creative Commons Zero "No rights reserved" data waiver (CC0 1.0 Public domain dedication). 

**Dataset 1: Data on 2016 massive open online courses about disease outbreaks, offered on learning platforms, retrieved from manual searches.** The dataset contains information on the learning platform, institution, course length, time required per week, language and availability of subtitles. DOI, 10.5256/f1000research.12639.d177854 ^6^.
