# Supplementary material for: Utility of massive open online courses (MOOCs) concerning outbreaks of emerging and reemerging diseases
Source: F1000Res. 2017 Dec 27;6:1699. Originally published 2017 Sep 18. [Version 2] doi: 10.12688/f1000research.12639.2 (PMC5717472; doi:10.12688/f1000research.12639.2)
Supplement: Peer review history

# Peer review: Referee response for version 1

**DOI:** 10.5256/f1000research.13685.r28591

**Published:** 2017-12-05

**Authors:** Shirley Ann Williams, Tharindu Liyanagunawardena

**Affiliations:** 1 School of Systems Engineering, University of Reading, Reading, UK; 2 University College of Estate Management, Reading, UK

This is an interesting research note identifying MOOCs relating to emerging and re-emerging diseases. The work in its current form is acceptable as a research note, and the conclusion that such MOOCs need to be available in more languages is an important point.

 If the authors are to extend their work there is scope for improvement:

1. The search window was very limited, it is possible that MOOCs that had completed would not be found. Adding MOOC aggregators (such as Class Central) to the search areas could have identified more courses, than those on offer in May 2017.
2. There are many barriers to accessing MOOCs, especially in developing countries, for example access to computers, connectivity, digital skills, as well as fluency in international languages. Consideration of all potential barriers will be important in future work.
3. The target audience of the MOOCs is not clear in this report, and there may be benefit in classifying courses that are aimed at health professionals and those aimed at the general public. Then considering whether there is  benefit in a direct linguistic translation for particular MOOCs.

We have read this submission. We believe that we have an appropriate level of expertise to confirm that it is of an acceptable scientific standard.

# Response

**Published:** 2017-12-19

**Authors:** Akram Hernández-Vásquez

**Affiliations:** Universidad Privada del Norte. Lima, Perú, Peru

Dear Dr. Williams and Dr. Liyanagunawardena

Thank you very much for your response. Regarding your comments:

1. About the use of MOOC aggregators, the authors used Class Central and MOOC List for both obtain the list of MOOC websites and for obtaining information about MOOCs completed before our time frame. Now, that information is included in methods:

From 1st May to 31st May 2017, we conducted a manual search on several learning platforms that offer MOOCs, including Coursera, edX, FutureLearn, Udacity, Miríada X, Alison, FUN.MOOC, Canvas Network, and University to find courses about disease outbreaks using the terms included in the list of WHO disease outbreaks from January 1st to December 31st, 2016 (Box 1). ***If the information about the course was not available on the learning platform that originally offered it, we use the information provided by MOOC aggregator platforms such as Class Central and MOOC List*** because Information about the learning platform, institution, course length, time required per week, language and subtitles availability for every course were collected and reported using frequencies in the case of categorical data and ranges for numerical data.

2. Thank you for your recommendation. Our study group is developing a survey to measure barriers to accessing and using MOOCs produced locally.

3. About the target population, all the courses were aimed at health professionals. Considering this, we have included this information in the results section:

We found seven courses about Ebola, two about Zika, three about the dynamics of epidemics and pandemics, and only one course about dengue, chikungunya, and malaria. The duration of the courses ranged from 2 to 10 weeks. 11 out of 13 courses were held only in English, with the possibility to select subtitles in English or other languages; there was one in English and Chinese and only one exclusively in Spanish. Most courses (5 out of 13) originated from to USA centers including Emory University, University of Pittsburgh, The Pennsylvania State University, Harvard University and University System of Maryland. The information provided with the courses included audiovisual material, papers, and self-assessments. ***All the courses were made for health-related professionals*** and presented information about epidemiology and lessons about the outbreaks and prevention activities for a possible new scenario of transmission of infectious diseases. The courses on Ebola, Zika, and chikungunya were released after the last outbreaks of these diseases, respectively.

# Peer review: Referee response for version 1

**DOI:** 10.5256/f1000research.13685.r26029

**Published:** 2017-09-29

**Authors:** Mohamed A Gouda

**Affiliations:** 1 Faculty of Medicine, Menoufia University, Shebin Al-Kom, Egypt

This is a nice short report from the study conducted by Bendezu-Quispe G *et al.* about the existence of MOOCs with focus on emerging and re-emerging diseases. The idea is simple, and so is the methodology. However, I have concern regarding the time frame selected for the study which was limited to the year 2016. Some of the outbreaks highlighted in the background started in earlier years and were terminated in 2016. I think that more valid and strong data could have been provided if there were no time limits thus allowing for exploration of MOOCs presented at the time of epidemics. Otherwise, authors have properly presented their data. I believe their work would be a good start for further exploration of this area.

I have read this submission. I believe that I have an appropriate level of expertise to confirm that it is of an acceptable scientific standard.

# Response

**Published:** 2017-12-19

**Authors:** Akram Hernández-Vásquez

**Affiliations:** Universidad Privada del Norte. Lima, Perú, Peru

Dear Dr. Gouda

Thank you for your comments regarding our Research note on MOOCs on emerging and reemerging diseases. About the time frame, in February 2016, WHO declared Zika’s outbreak a public health emergency of international concern. Based on that announcement, we conducted a search about MOOCs of Zika. We expected that both public and academia concern about this disease will impact the number of MOOCs developed about this topic. However, the presence of outbreaks of diseases such as chikungunya and Oropuche during 2016, expanded our research scope. The 2016 WHO’s list of diseases outbreaks included: Ebola, viral hemorrhagic fevers, hemorrhagic fever syndrome, Zika, Chikungunya,  Lassa, MERS-CoV, Middle East respiratory syndrome coronavirus, Yellow fever, Human infection with avian influenza, Oropouche virus, Rift Valley fever and Wild polio and vaccine derived polio. This list includes a vast number of diseases that were also included in the annual lists of outbreaks published by WHO before 2016. Hence, it is not expected that a significant increase in the number of MOOCs after increasing the list of outbreaks before 2016.
